# The genome sequence of the European plaice, *Pleuronectes platessa* (Linnaeus, 1758)

**DOI:** 10.12688/wellcomeopenres.19397.1

**Published:** 2023-08-30

**Authors:** Rachel Brittain, Mitchell Brenen, Sean McTierney, Patrick Adkins, Robert Mrowicki, Joanna Harley, Marine Biological Association Genome Acquisition Lab, Darwin Tree of Life Barcoding collective, Wellcome Sanger Institute Tree of Life programme, Wellcome Sanger Institute Scientific Operations: DNA Pipelines collective, Tree of Life Core Informatics collective, Darwin Tree of Life Consortium

**Affiliations:** 1 The Marine Biological Association, Plymouth, England, UK

**Keywords:** Pleuronectes platessa, European plaice, genome sequence, chromosomal; Pleuronectiformes

## Abstract

We present a genome assembly from an individual *Pleuronectes platessa*(the European plaice; Chordata; Actinopteri; Pleuronectiformes; Pleuronectidae). The genome sequence is 687.4 megabases in span. Most of the assembly is scaffolded into 24 chromosomal pseudomolecules. The mitochondrial genome has also been assembled and is 17.4 kilobases in length.

## Species taxonomy

Eukaryota; Metazoa; Chordata; Craniata; Vertebrata; Euteleostomi; Actinopterygii; Neopterygii; Teleostei; Neoteleostei; Acanthomorphata; Carangaria; Pleuronectiformes; Pleuronectoidei; Pleuronectidae; *Pleuronectes*; *Pleuronectes platessa* (Linnaeus, 1758) (NCBI:txid8262).

## Background

The European plaice *Pleuronectes platessa* (Linnaeus, 1758), is common species of flatfish belonging to the family Pleuronectidae. Pleuronectidae have compressed, oval shaped bodies with both eyes on the right side and are widely distributed in cool temperate waters. *P. platessa* is a bottom-living fish found on sand, mud, and gravel substrates down to 200 m, but they are more common at depths ranging from 10 to 50 m. Juveniles live in shallow water, frequenting estuaries and the intertidal zone and will move into deeper water when they attain a greater size at approximately 2 years. The species identified by its conspicuous red/orange spots on the dorsal side of its body and has a series of bony knobs that run in a curved line from the eyes back to the lateral line. Adult fish can grow up to 90 cm long, with most adults reaching lengths of 50 to 60 cm ( Wheeler, 1978).

*P. platessa* occur from the Barents Sea and Iceland to southern Spain and the western Mediterranean, but are most common in the seas surrounding the British Isles ( Dipper, 2022). It is a commercially important species in the north-east Atlantic region and is the most economically important species of flatfish for European fisheries ( Madsen *et al.*, 2013; Prellezo & Carvahlo, 2020) with several stocks around the UK including the eastern English Channel, western English Channel, North Sea, Celtic Sea, and Irish Sea ( Ellis *et al.*, 2012).

Unsustainable levels of exploitation during the 1970s and 1980s reduced *P. platessa* spawning stock biomass to critical levels. Since then, fishing pressure has reduced, allowing spawning stock biomass to increase over the last 5–10 years in all stocks and is now listed as of Least Concern on the IUCN Red List due to the population trend increasing ( Freyhof, 2014).

## Genome sequence report

**Figure 1.**
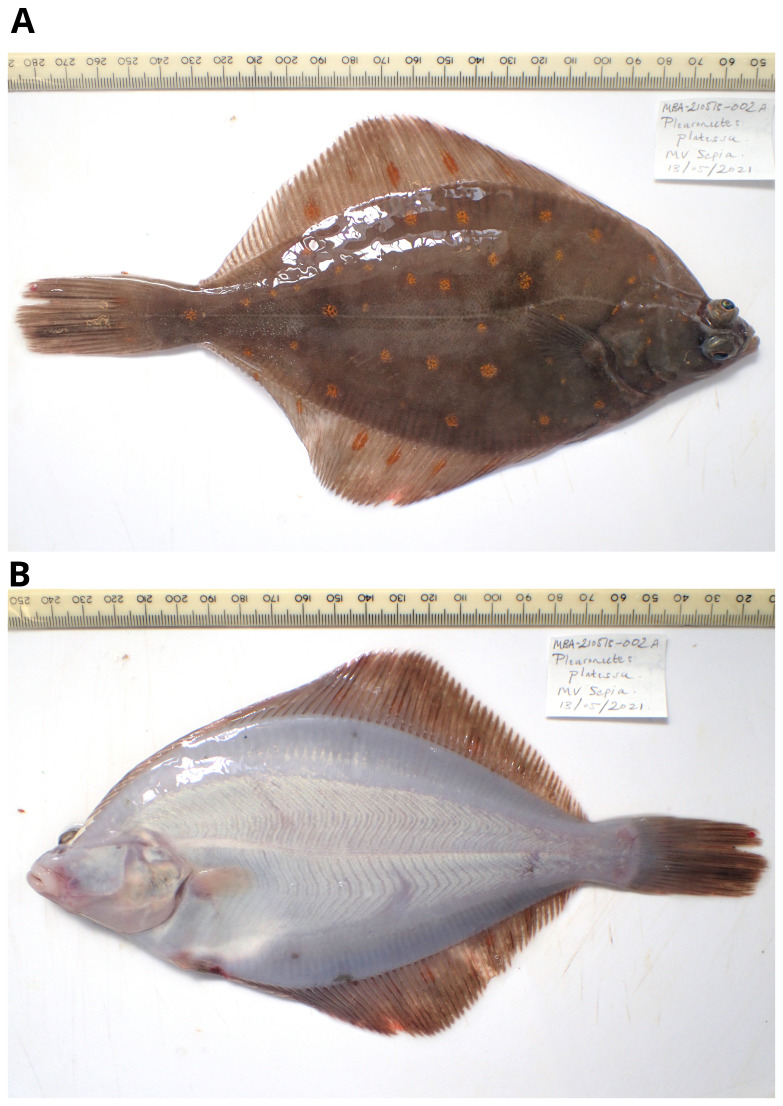
Photographs of the *Pleuronectes platessa* (fPlePla1) specimen used for genome sequencing. **A**. right side, **B**. left side.

The genome was sequenced from one *Pleuronectes platessa* ( Figure 1) collected from Looe Ground, Cornwall, UK (latitude 50.28, longitude –4.24). A total of 35-fold coverage in Pacific Biosciences single-molecule HiFi long reads was generated. Primary assembly contigs were scaffolded with chromosome conformation Hi-C data. Manual assembly curation corrected 103 missing joins or mis-joins and removed 32 haplotypic duplications, reducing the scaffold number by 17.97%, and increasing the scaffold N50 by 0.82%.

**Figure 2.**
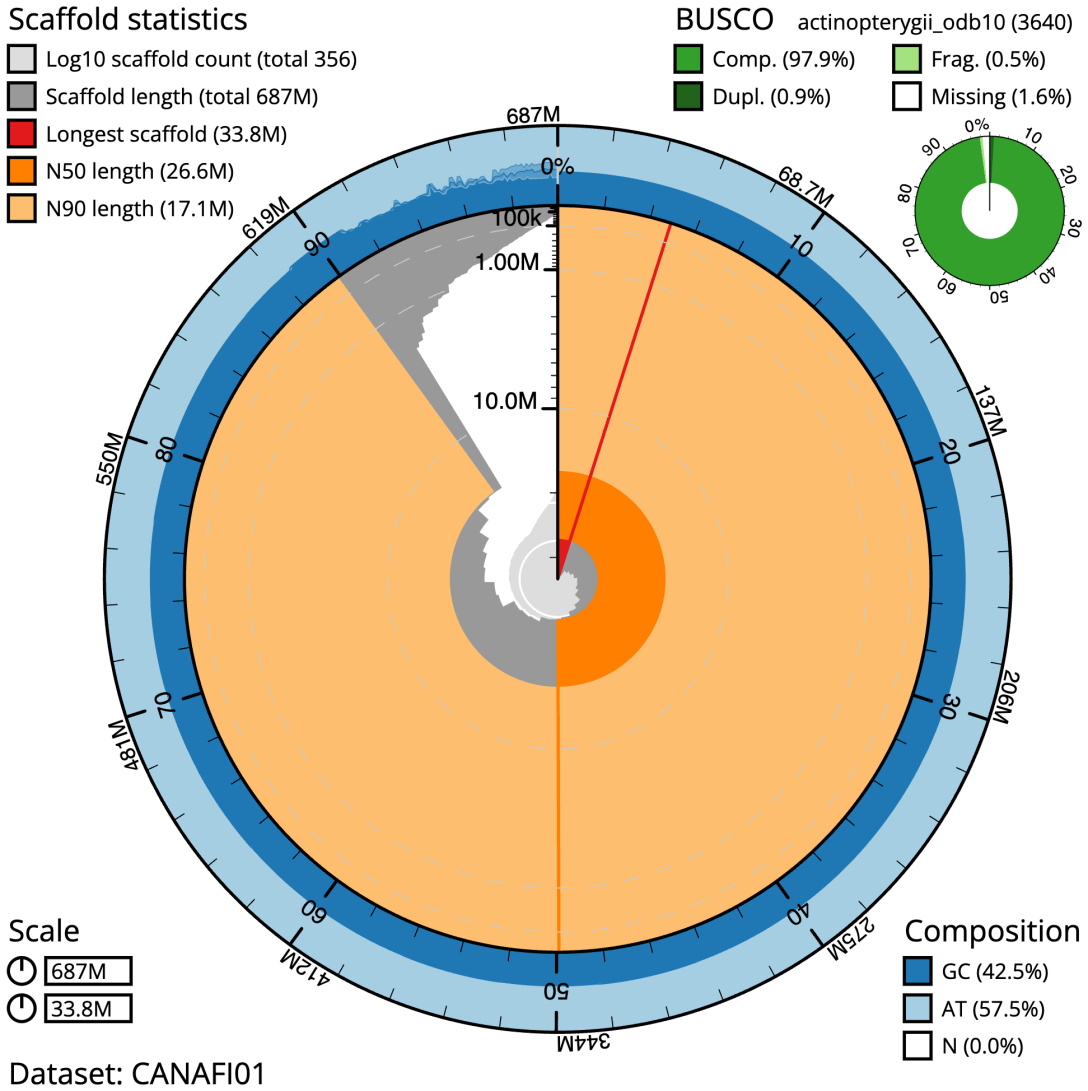
Genome assembly of *Pleuronectes platessa*, fPlePla1.1: metrics. The BlobToolKit Snailplot shows N50 metrics and BUSCO gene completeness. The main plot is divided into 1,000 size-ordered bins around the circumference with each bin representing 0.1% of the 687,405,470 bp assembly. The distribution of scaffold lengths is shown in dark grey with the plot radius scaled to the longest scaffold present in the assembly (33,765,178 bp, shown in red). Orange and pale-orange arcs show the N50 and N90 scaffold lengths (26,648,505 and 17,067,268 bp), respectively. The pale grey spiral shows the cumulative scaffold count on a log scale with white scale lines showing successive orders of magnitude. The blue and pale-blue area around the outside of the plot shows the distribution of GC, AT and N percentages in the same bins as the inner plot. A summary of complete, fragmented, duplicated and missing BUSCO genes in the actinopterygii_odb10 set is shown in the top right. An interactive version of this figure is available at https://blobtoolkit.genomehubs.org/view/fPlePla1.1/dataset/CANAFI01/snail.

The final assembly has a total length of 687.4 Mb in 356 sequence scaffolds with a scaffold N50 of 26.6 Mb ( Table 1). Most (91.24%) of the assembly sequence was assigned to 24 chromosomal-level scaffolds. Chromosome-scale scaffolds confirmed by the Hi-C data are named in order of size ( Figure 2– Figure 5; Table 2). While not fully phased, the assembly deposited is of one haplotype. Contigs corresponding to the second haplotype have also been deposited. The mitochondrial genome was also assembled and can be found as a contig within the multifasta file of the genome submission.

**Table 1.** Genome data for *Pleuronectes platessa*, fPlePla1.1.

Project accession data		
Assembly identifier	fPlePla1.1	
Species	*Pleuronectes platessa*	
Specimen	fPlePla1	
NCBI taxonomy ID	8262	
BioProject	PRJEB56054	
BioSample ID	SAMEA13853390	
Isolate information	fPlePla1, muscle tissue	
Assembly metrics *		*Benchmark*
Consensus quality (QV)	54.9	*≥ 50*
*k*-mer completeness	99.99%	*≥ 95%*
BUSCO **	C:97.9%[S:97.0%,D:0.9%], F:0.5%,M:1.6%,n:3,640	*C ≥ 95%*
Percentage of assembly mapped to chromosomes	91.24%	*≥ 95%*
Sex chromosomes	-	*localised homologous pairs*
Organelles	Mitochondrial genome assembled	*complete single alleles*
Raw data accessions		
PacificBiosciences SEQUEL II	ERR10224925	
Hi-C Illumina	ERR10297816	
PolyA RNA-Seq Illumina	ERR10908603	
Genome assembly		
Assembly accession	GCA_947347685.1	
*Accession of alternate haplotype*	GCA_947347695.1	
Span (Mb)	687.4	
Number of contigs	1,428	
Contig N50 length (Mb)	1.5	
Number of scaffolds	356	
Scaffold N50 length (Mb)	26.6	
Longest scaffold (Mb)	33.8	

* Assembly metric benchmarks are adapted from column VGP-2020 of “Table 1: Proposed standards and metrics for defining genome assembly quality” from ( Rhie *et al.*, 2021).

** BUSCO scores based on the actinopterygii_odb10 BUSCO set using v5.3.2. C = complete [S = single copy, D = duplicated], F = fragmented, M = missing, n = number of orthologues in comparison. A full set of BUSCO scores is available at https://blobtoolkit.genomehubs.org/view/fPlePla1.1/dataset/CANAFI01/cumulative.

**Figure 3.**
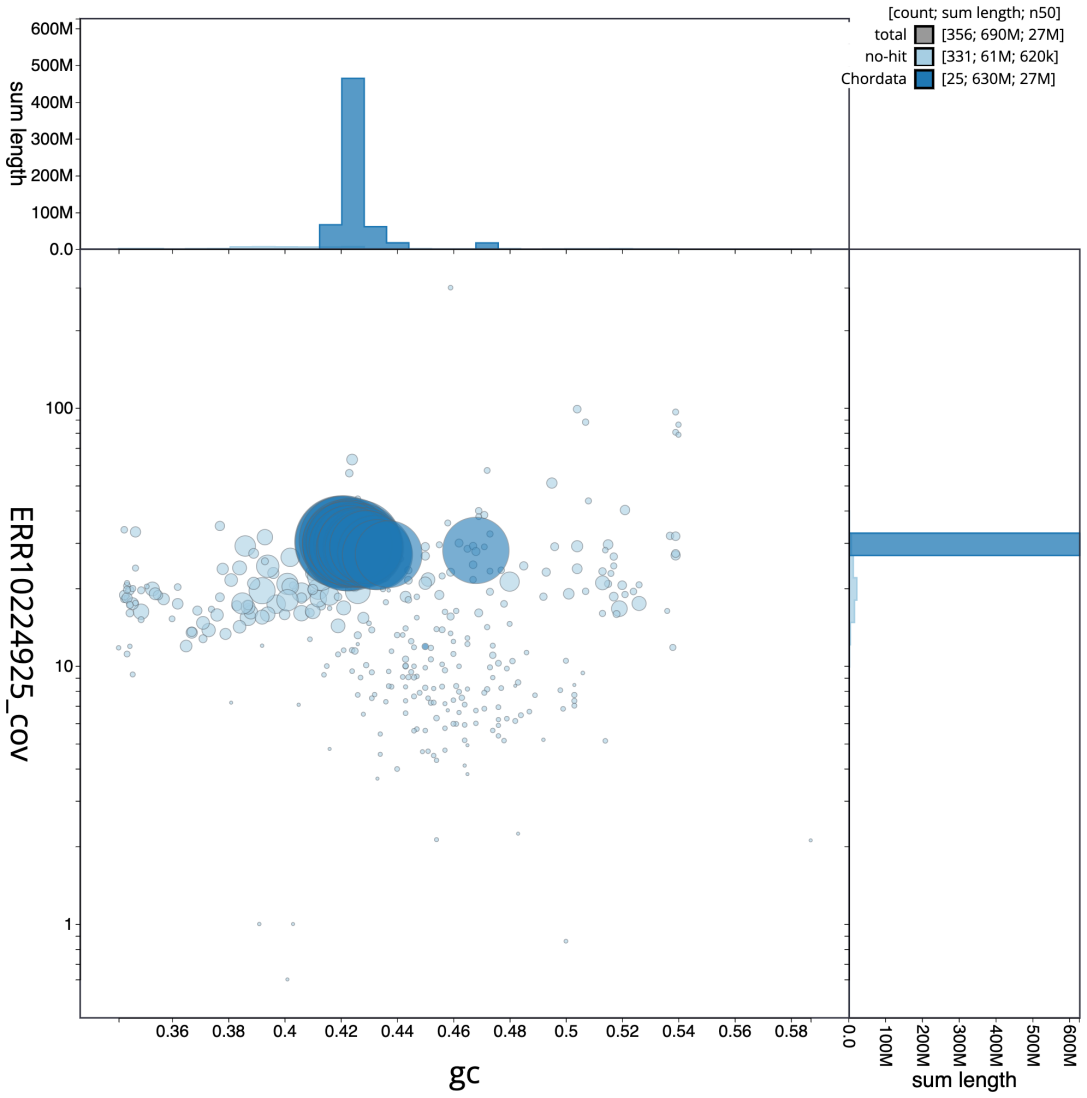
Genome assembly of *Pleuronectes platessa*, fPlePla1.1: GC coverage. BlobToolKit GC-coverage plot. Scaffolds are coloured by phylum. Circles are sized in proportion to scaffold length. Histograms show the distribution of scaffold length sum along each axis. An interactive version of this figure is available at https://blobtoolkit.genomehubs.org/view/fPlePla1.1/dataset/CANAFI01/blob.

**Figure 4.**
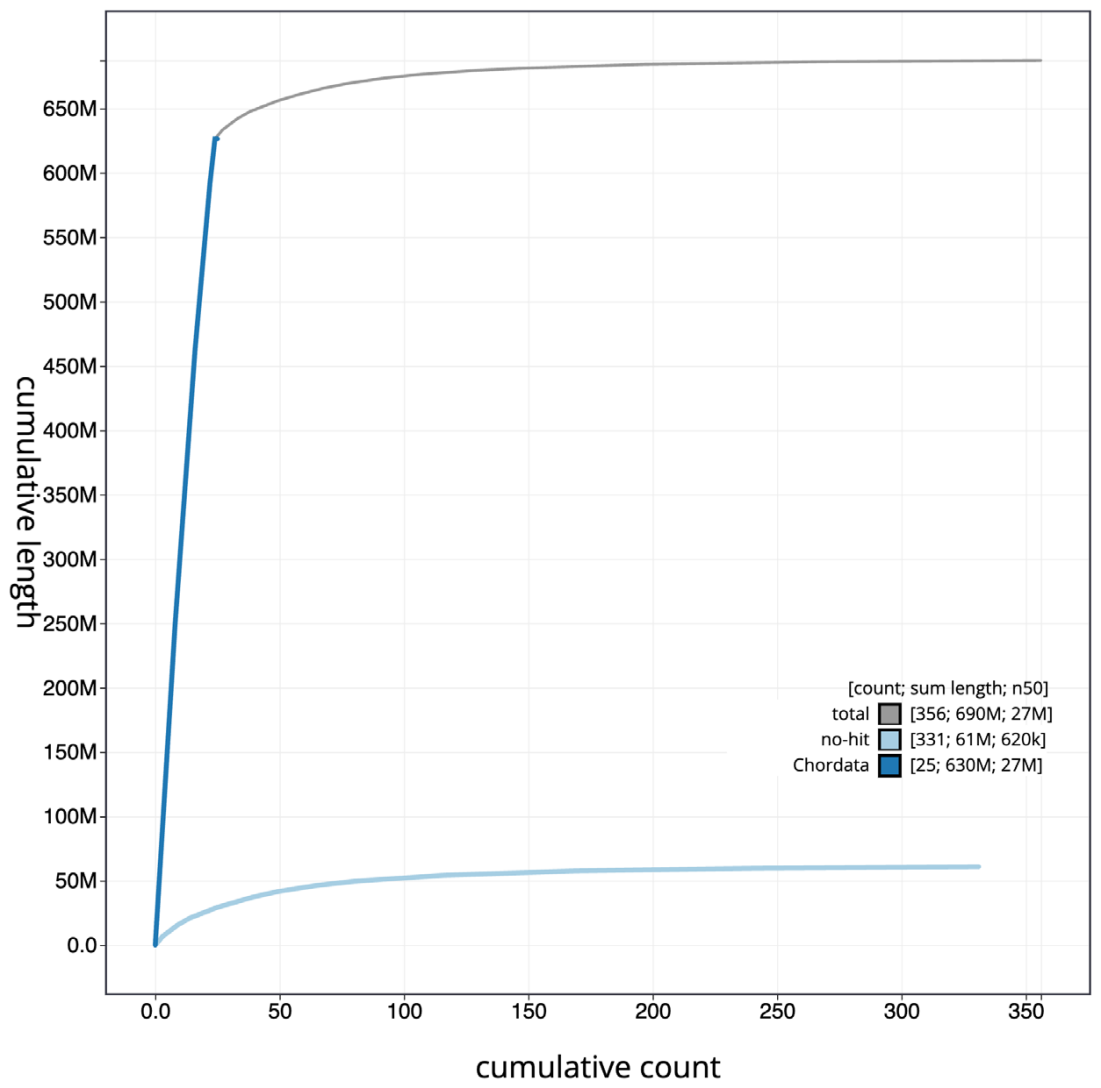
Genome assembly of *Pleuronectes platessa*, fPlePla1.1: cumulative sequence. BlobToolKit cumulative sequence plot. The grey line shows cumulative length for all scaffolds. Coloured lines show cumulative lengths of scaffolds assigned to each phylum using the buscogenes taxrule. An interactive version of this figure is available at https://blobtoolkit.genomehubs.org/view/fPlePla1.1/dataset/CANAFI01/cumulative.

**Figure 5.**
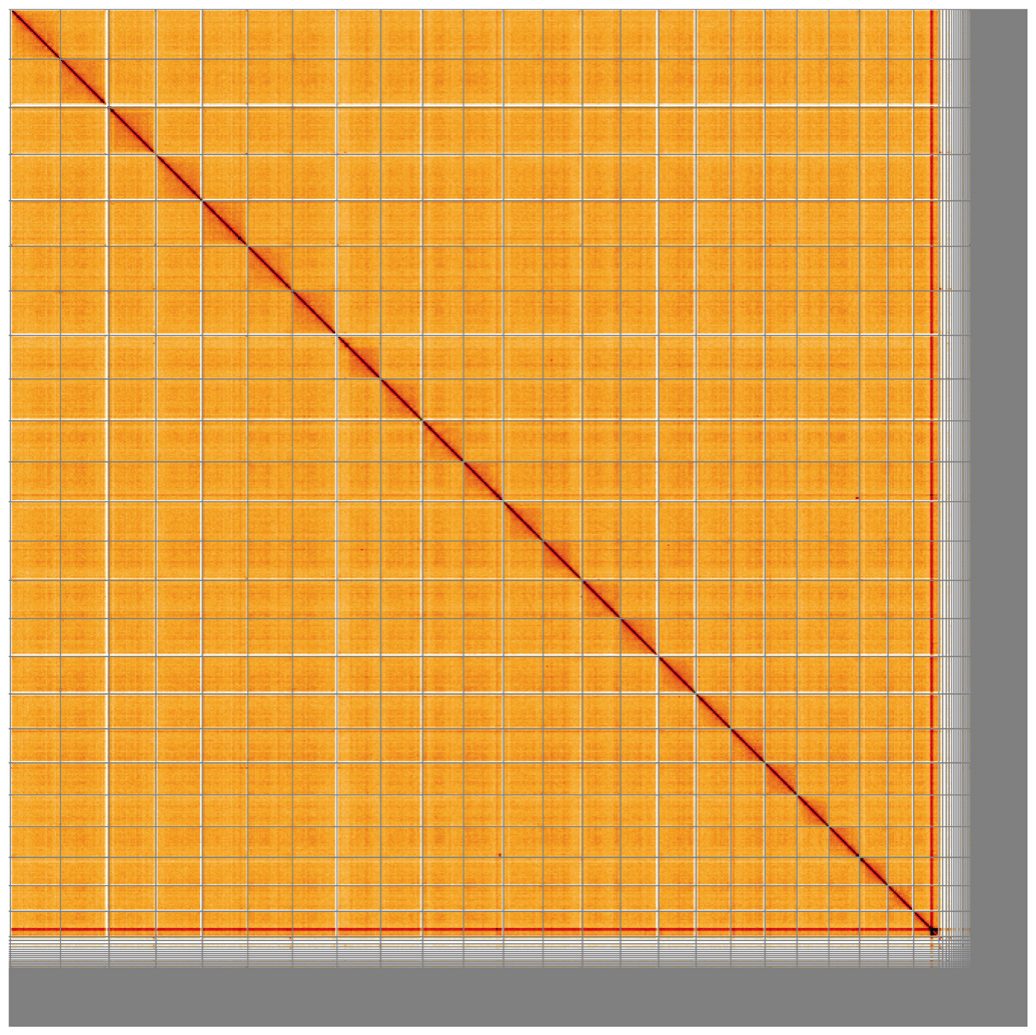
Genome assembly of *Pleuronectes platessa*, fPlePla1.1: Hi-C contact map. Hi-C contact map of the fPlePla1.1 assembly, visualised using HiGlass. Chromosomes are shown in order of size from left to right and top to bottom. An interactive version of this figure may be viewed at https://genome-note-higlass.tol.sanger.ac.uk/l/?d=MfSPpN31RZmJxGNO9eKGuQ.

**Table 2.** Chromosomal pseudomolecules in the genome assembly of *Pleuronectes platessa*, fPlePla1.

INSDC accession	Chromosome	Size (Mb)	GC%
OX374648.1	1	33.77	42
OX374649.1	2	32.75	42
OX374650.1	3	31.71	42.3
OX374651.1	4	31.25	42.1
OX374652.1	5	30.71	42.2
OX374653.1	6	30.13	42.6
OX374654.1	7	30.02	42.4
OX374655.1	8	29.49	42.2
OX374656.1	9	28.27	42.3
OX374657.1	10	27.62	42.4
OX374658.1	11	26.96	42.7
OX374659.1	12	26.65	42.1
OX374660.1	13	26.54	42.5
OX374661.1	14	25.77	42.2
OX374662.1	15	25.57	42.3
OX374663.1	16	25.33	42.7
OX374664.1	17	23.44	42.5
OX374665.1	18	23.1	42.8
OX374666.1	19	21.67	42.5
OX374667.1	20	21.4	43.2
OX374668.1	21	20.73	42.9
OX374669.1	22	19.17	43.3
OX374670.1	23	17.31	43.7
OX374671.1	24	17.07	46.8
OX374672.1	MT	0.02	46.2
-	unplaced	60.97	41.7

The estimated Quality Value (QV) of the final assembly is 54.9 with *k*-mer completeness of 99.99%, and the assembly has a BUSCO v5.3.2 completeness of 97.9% (single = 97.0%, duplicated = 0.9%), using the actinopterygii_odb10 reference set ( *n* = 3,640).

Metadata for specimens, spectral estimates, sequencing runs, contaminants and pre-curation assembly statistics can be found at https://links.tol.sanger.ac.uk/species/8262.

## Methods

### Sample acquisition and nucleic acid extraction

A *Pleuronectes platessa* specimen (specimen no. MBA-210513–002A, ToLID fPlePla1) was collected from Looe Ground, Cornwall, UK (latitude 50.28, longitude –4.24) on 13 May 2021. The specimen was taken from its habitat of broken shell and muddy sand by Sean Mctierney, Rachel Brittain and Mitchell Brenen (Marine Biological Association) using an otter trawl from the MV Sepia. The specimen was identified by Robert Mrowicki, Patrick Adkins, Joanna Harley and Rachel Brittain (Marine Biological Association) based on gross morphology. The fish was first anesthetised and then overdosed using Aquased (2-phenoxyethanol). Destruction of the brain was used as a secondary method to ensure the animal was deceased before tissue sampling took place as in accordance with Schedule 1 methodology under the home office licence. Samples taken from the animal were preserved in liquid nitrogen.

DNA was extracted at the Tree of Life laboratory, Wellcome Sanger Institute (WSI). The fPlePla1 sample was weighed and dissected on dry ice with tissue set aside for Hi-C sequencing. Muscle tissue was cryogenically disrupted to a fine powder using a Covaris cryoPREP Automated Dry Pulveriser, receiving multiple impacts. High molecular weight (HMW) DNA was extracted using the Qiagen MagAttract HMW DNA extraction kit. HMW DNA was sheared into an average fragment size of 12–20 kb in a Megaruptor 3 system with speed setting 30. Sheared DNA was purified by solid-phase reversible immobilisation using AMPure PB beads with a 1.8X ratio of beads to sample to remove the shorter fragments and concentrate the DNA sample. The concentration of the sheared and purified DNA was assessed using a Nanodrop spectrophotometer and Qubit Fluorometer and Qubit dsDNA High Sensitivity Assay kit. Fragment size distribution was evaluated by running the sample on the FemtoPulse system.

RNA was extracted from muscle tissue of fPlePla1 in the Tree of Life Laboratory at the WSI using TRIzol, according to the manufacturer’s instructions. RNA was then eluted in 50 μl RNAse-free water and its concentration assessed using a Nanodrop spectrophotometer and Qubit Fluorometer using the Qubit RNA Broad-Range (BR) Assay kit. Analysis of the integrity of the RNA was done using Agilent RNA 6000 Pico Kit and Eukaryotic Total RNA assay.

### Sequencing

Pacific Biosciences HiFi circular consensus and 10X Genomics read cloud DNA sequencing libraries were constructed according to the manufacturers’ instructions. Poly(A) RNA-Seq libraries were constructed using the NEB Ultra II RNA Library Prep kit. DNA and RNA sequencing were performed by the Scientific Operations core at the WSI on Pacific Biosciences SEQUEL II (HiFi) and Illumina NovaSeq 6000 (RNA-Seq) instruments. Hi-C data were also generated from muscle tissue of fPlePla1 using the Arima2 kit and sequenced on the Illumina NovaSeq 6000 instrument.

### Genome assembly, curation and evaluation

Assembly was carried out with Hifiasm ( Cheng *et al.*, 2021) and haplotypic duplication was identified and removed with purge_dups ( Guan *et al.*, 2020). The assembly was scaffolded with Hi-C data ( Rao *et al.*, 2014) using YaHS ( Zhou *et al.*, 2023). The assembly was checked for contamination as described previously ( Howe *et al.*, 2021). Manual curation was performed using HiGlass ( Kerpedjiev *et al.*, 2018) and Pretext ( Harry, 2022). The mitochondrial genome was assembled using MitoHiFi ( Uliano-Silva *et al.*, 2022), which runs MitoFinder ( Allio *et al.*, 2020) or MITOS ( Bernt *et al.*, 2013) and uses these annotations to select the final mitochondrial contig and to ensure the general quality of the sequence. To evaluate the assembly, MerquryFK was used to estimate consensus quality (QV) scores and *k*-mer completeness ( Rhie *et al.*, 2020). The genome was analysed within the BlobToolKit environment ( Challis *et al.*, 2020) and BUSCO scores ( Manni *et al.*, 2021; Simão *et al.*, 2015) were calculated. Table 3 contains a list of software tool versions and sources.

**Table 3.** Software tools: versions and sources.

Software tool	Version	Source
BlobToolKit	4.0.7	https://github.com/ blobtoolkit/blobtoolkit
BUSCO	5.3.2	https://gitlab.com/ezlab/ busco
Hifiasm	0.16.1-r375	https://github.com/ chhylp123/hifiasm
HiGlass	1.11.6	https://github.com/higlass/ higlass
Merqury	MerquryFK	https://github.com/ thegenemyers/MERQURY.FK
MitoHiFi	2	https://github.com/ marcelauliano/MitoHiFi
PretextView	0.2	https://github.com/wtsi- hpag/PretextView
purge_dups	1.2.3	https://github.com/dfguan/ purge_dups
YaHS	yahs-1.1.91eebc2	https://github.com/c-zhou/ yahs

### Wellcome Sanger Institute – Legal and Governance

The materials that have contributed to this genome note have been supplied by a Darwin Tree of Life Partner.

The submission of materials by a Darwin Tree of Life Partner is subject to the **‘Darwin Tree of Life Project Sampling Code of Practice’**, which can be found in full on the Darwin Tree of Life website here. By agreeing with and signing up to the Sampling Code of Practice, the Darwin Tree of Life Partner agrees they will meet the legal and ethical requirements and standards set out within this document in respect of all samples acquired for, and supplied to, the Darwin Tree of Life Project.

Further, the Wellcome Sanger Institute employs a process whereby due diligence is carried out proportionate to the nature of the materials themselves, and the circumstances under which they have been/are to be collected and provided for use. The purpose of this is to address and mitigate any potential legal and/or ethical implications of receipt and use of the materials as part of the research project, and to ensure that in doing so we align with best practice wherever possible.

The overarching areas of consideration are:

- Ethical review of provenance and sourcing of the material
- Legality of collection, transfer and use (national and international)

Each transfer of samples is further undertaken according to a Research Collaboration Agreement or Material Transfer Agreement entered into by the Darwin Tree of Life Partner, Genome Research Limited (operating as the Wellcome Sanger Institute), and in some circumstances other Darwin Tree of Life collaborators.

## Data Availability

European Nucleotide Archive: Pleuronectes platessa (European plaice). Accession number PRJEB56054; https://identifiers.org/ena.embl/PRJEB56054. ( Wellcome Sanger Institute, 2022) The genome sequence is released openly for reuse. The *Pleuronectes platessa* genome sequencing initiative is part of the Darwin Tree of Life (DToL) project. All raw sequence data and the assembly have been deposited in INSDC databases. The genome will be annotated using available RNA-Seq data and presented through the Ensembl pipeline at the European Bioinformatics Institute. Raw data and assembly accession identifiers are reported in Table 1.

## Author information

Members of the Marine Biological Association Genome Acquisition Lab are listed here: https://doi.org/10.5281/zenodo.4783605.

Members of the Darwin Tree of Life Barcoding collective are listed here: https://doi.org/10.5281/zenodo.4893703.

Members of the Wellcome Sanger Institute Tree of Life programme are listed here: https://doi.org/10.5281/zenodo.4783585.

Members of Wellcome Sanger Institute Scientific Operations: DNA Pipelines collective are listed here: https://doi.org/10.5281/zenodo.4790455.

Members of the Tree of Life Core Informatics collective are listed here: https://doi.org/10.5281/zenodo.5013541.

Members of the Darwin Tree of Life Consortium are listed here: https://doi.org/10.5281/zenodo.4783558.
