# Supplementary material for: The genome sequence of the European plaice, Pleuronectes platessa (Linnaeus, 1758)
Source: Wellcome Open Res. 2023 Aug 30;8:367. [Version 1] doi: 10.12688/wellcomeopenres.19397.1 (PMC10997986; doi:10.12688/wellcomeopenres.19397.1)
Supplement: Peer review history

# Reviewer report: Reviewer response for version 1

**DOI:** 10.21956/wellcomeopenres.21486.r75252

**Published:** 2024-04-05

**Authors:** Alan Le Moan

**Affiliations:** 1 University of Gothenburg, Strömstad, Sweden

This article presents a reference genome for the European plaice ( *Pleuronectes platessa*). This genome is spited across 24 chromosome (91% of the total genome) and 332 unplaced contigs. This new genomic resource will be relevant for many researchers working on this commercially important species. On a side note: There is already published work on population genomics of the European plaice showing that two large chromosomal rearrangements (two putative inversions) segregate in northern plaice populations (North Sea, Baltic Sea, Barents Sea, and Iceland) and distinguish different plaice populations (see [1] and [2]). I cannot see evidence for these inversions being polymorphic in the individual sequenced for the current assembly. However, I think this information would be worth mentioning somewhere in the note in case people uses this genome in future genomic work.

 I also noticed one surprising result that could benefit from some clarification in the text. In the BlobToolKit GC-coverage plot, there is a quite large variation of GC content across the unplaced contig that is somewhat surprising. I was wondering to what extent this could be driven by contamination? I don’t think other genomes have this wide range of variation the other notes.

 All of my comments are very minor details, and otherwise, the note is very well written and presented.

Are sufficient details of methods and materials provided to allow replication by others?

Yes

Is the rationale for creating the dataset(s) clearly described?

Yes

Are the datasets clearly presented in a useable and accessible format?

Yes

Are the protocols appropriate and is the work technically sound?

Yes

Reviewer Expertise:

Population genomics

I confirm that I have read this submission and believe that I have an appropriate level of expertise to confirm that it is of an acceptable scientific standard.

## References

[1] : Evolution at two time frames: ancient structural variants involved in post-glacial divergence of the European plaice (Pleuronectes platessa). *Heredity (Edinb)* .2021;126(4) : 10.1038/s41437-020-00389-3 668-683 10.1038/s41437-020-00389-3 33531657 PMC8115344

[2] : The role of genomic signatures of directional selection and demographic history in the population structure of a marine teleost with high gene flow. *Ecol Evol* .2022;12(12) : 10.1002/ece3.9602 e9602 10.1002/ece3.9602 36514551 PMC9731920

# Reviewer report: Reviewer response for version 1

**DOI:** 10.21956/wellcomeopenres.21486.r70057

**Published:** 2023-11-29

**Authors:** Stephan Koblmüller

**Affiliations:** 1 University of Graz, Graz, Austria

The authors present a Data Note on a complete genome assembly for the European plaice, *Pleuronectes platessa*, a commercially important fish species (hence, it would be great if the authors could add a sentence about the relevance of this high quality reference genome for population/conservation genomics and stock management of this species). As a "side-product" they also present the mitochondrial genome assembly. The genome is sequenced from a single individual. In terms of quality, this genome resource is quite good, judging from the summary statistics provided in this Data Note.

 This Data Note is straightforward and well written with a clear description of the results and methods used (including bioinformatics software).

Are sufficient details of methods and materials provided to allow replication by others?

Yes

Is the rationale for creating the dataset(s) clearly described?

Yes

Are the datasets clearly presented in a useable and accessible format?

Yes

Are the protocols appropriate and is the work technically sound?

Yes

Reviewer Expertise:

phylogenetics, phylogeography, population genetics of a wide range of taxa

I confirm that I have read this submission and believe that I have an appropriate level of expertise to confirm that it is of an acceptable scientific standard.
